# Inclusion and diversity within medical education: a focus group study of students’ experiences

**DOI:** 10.1186/s12909-023-04036-3

**Published:** 2023-01-25

**Authors:** Anne-Roos Verbree, Ulviye Isik, Jeroen Janssen, Gönül Dilaver

**Affiliations:** 1 Education Center, University Medical Center Utrecht, Utrecht, Netherlands; 2 Faculty of Social and Behavioral Sciences, Utrecht University, Utrecht, Netherlands

**Keywords:** Medical education, Medical curriculum, Diversity, Inclusion, Inclusive learning environment, Sense of belonging, Student experience

## Abstract

**Background/introduction:**

As patient populations become more diverse, it is imperative that future physicians receive proper training in order to provide the best quality of care. This study examines medical students' perceptions of how prepared they are in dealing with a diverse population and assesses how included and supported the students felt during their studies.

**Methods:**

Four semi-structured focus groups were held with medical students across all years of the medical study program of a Dutch university. Focus group transcripts were analyzed thematically.

**Results:**

Students’ experiences could be categorized as follows: (1) (Minority) identities and personal motivations, (2) Understanding of diversity and an inclusive learning environment, (3) Diversity in education, (4) Experiences of exclusion, (5) Experiences of inclusion, and (6) Lack of awareness. The key findings from the focus groups were that students perceived a lack of diversity and awareness in medical education and were convinced of the need to incorporate diversity to a greater extent and were personally motivated to contribute to incorporating diversity in the curriculum. Students also shared exclusion experiences such as stereotypes and prejudices but also some inclusion experiences such as feelings of belonging.

**Conclusion:**

Based on our findings, it is recommended that medical schools incorporate diversity education into their curriculum so that health professionals can provide the best quality of care for their diverse patient populations. This education should also ensure that all students feel included in their medical education program.

**Supplementary Information:**

The online version contains supplementary material available at 10.1186/s12909-023-04036-3.

A skilled medical workforce that will promote the health of all people is critical to the health of any nation [1]. As society is diverse in many respects, including gender/sex, ethnicity, nationality, age, religion, socioeconomic status, sexual orientation, and disability [2], future medical practitioners must be prepared to meet the needs of this diverse patient population [3]. This is even more relevant given the evidence that patients with underrepresented ethnic backgrounds and lower socioeconomic positions tend to receive lower quality of care than other patients and that they experience greater morbidity and mortality from various chronic diseases than nonminorities [4].

Health care professionals can play a significant role in reducing the enormous disparities in health in an increasingly complex and diverse world today [5]. Therefore, it is the duty of medical education to prepare future health care professionals to practice medicine in this context [6, 7]. Dharamsi et al. (2011) frame this as developing the social responsibility of health care professionals: “ensuring, protecting, and contributing to the collective welfare of society. It is also about choosing to contribute to the common good rather than being legislated to do it.” (p. 1110) [5].

If health care professionals are called on to practice social responsibility throughout their careers, what skills do they need to develop to exercise that responsibility, and how can they be assisted in that? [5]. Graduates need the skills that will be required to work in today’s society which are different from those required of graduates in the past [8]. Medical students need skills to work in diverse teams and to work with diverse patients and communities in order to understand and address the population’s health needs [7]. This is needed to ensure that health care professionals are able to provide optimal care to all patients, to contribute to expanding health care access for the underserved, and to foster research in neglected areas of societal need [9]. However, it is not clear to what extent current medical education programs prepare students to take on this social responsibility in their careers as health care professionals. Moreover, relatively little research has been done into medical students’ views on the extent to which their education prepares them to work as a health professional in a diverse society as well as into their experiences of inclusion and exclusion in medical education, which points to the need of this being examined in the medical classroom [10].

The few studies that investigated medical students’ perceptions of the curriculum found that students generally do not feel prepared to work in a (culturally) diverse society as knowledge about difference, bias, resilience, and more is lacking in their education. Kai, Bridgewater, and Spencer (2001) conducted one of these studies and examined medical students’ perceptions and perceived training needs in relation to cultural and ethnic diversity in health care [11]. They found that most students experienced inadequate consideration of cultural and ethnic diversity in their training but at the same time, students have the willingness to learn more about those forms of diversity in health care. This is similar to the findings from a survey among a large sample of medical students about their preparedness and skillfulness in providing care to patients from diverse cultures that showed most medical students did not feel adequately prepared to provide care to diverse patients by their final year of education [12]. Also, students from a U.S. medical school pointed to gaps they observed in the curriculum and advocated for the establishment of a comprehensive curriculum that would adequately prepare them to work with diverse populations [13]. In short, in the traditional medical culture, diversity does not seem a central aspect of health care.

Including diversity(-related issues) in medical education may not only help future physicians to recognize patients as individuals with life stories that are affected by cultural forces, bias, discrimination, and resilience, but – in a similar vein – it may also contribute to medical students feeling respected and recognized as individuals with life experiences that are intimately related to ethnicity, gender, sexual orientation, socioeconomic status, and other characteristics [14]. The study of Mayhew, Grunwald, and Dey (2005) showed that that including diversity-related issues in the curriculum is important to make diverse students feel welcome and to bolster their sense of belonging as members of the campus community [15]. Sense of belonging in medical education, which is defined as the experience of being accepted, included, and valued by others [16], is important for a range of student outcomes including intentions to persist [17], academic and social adjustment to college, quality of experience at college, and academic achievement [18]. There appears to be little awareness of its importance in undergraduate health professions’ education, and research into students’ belonging in medical education is scarce [19]. The scarce research that has been done shows that minority students’ negative experiences, such as having poor relationships with majority students, have a negative influence on their sense of belonging in medical education [20, 21].

## Present study

It is the responsibility of medical schools to educate students to become health care professionals with competences to provide quality care to a great diversity of patients [5, 22]. It is also important to ensure students feel included and welcome in their programs [15]. The questions we must ask are: does current medical education prepare students to become health care professionals who can be effective with a range of populations [10], and do students perceive they are adequately prepared to provide care for a diverse patient population? Moreover, do diverse students feel represented in their curriculum and included and at home in their programs? The objective of this study was to gain insight into students’ perceptions on and experiences with diversity and inclusion in medical education in order to make it more inclusive. To answer the research questions, we conducted focus groups with medical students about their experiences of diversity within the medical curriculum, their experiences with inclusion and exclusion, their sense of belonging in medical education, and what they think can be improved within the curriculum and their education.

The results from this study provide insights into potential starting points on how to create an inclusive learning environment and foster diversity-sensitivity in the medical curriculum. Such insight is needed as it may foster students’ sense of belonging in medical education, which positively impacts their performance, psychological and academic adjustment, wellbeing, and other student outcomes [18, 19, 23]. In addition, it could lead to change in medical programs, which eventually may have a positive influence on the culture of the medical system [24]. The knock-on effect could contribute to positive change in the lives of patients, communities, and eventually health policy and health systems [5]. This transformation in medical education is the starting point of the development of socially responsible health care professionals who are equipped to practice medicine in a diverse and complex world.

## Method

This qualitative focus group study using thematic analysis was conducted at the University Medical Center Utrecht (UMCU), the Netherlands. The UMCU states on diversity and inclusion: “Inclusive and accessible education, with attention to diversity, affects all students. it builds bridges between students, challenges them to think about their future profession in a complex way, and reduces the risk of stereotypical thinking. The inclusive mindset our students develop in this way is a valuable part of their professional identity, which will eventually benefit health care too.” (https://www.umcutrecht.nl/nl/diversiteit-en-inclusie). Semi-structured focus groups with medical students provided valuable insights into students’ experiences and thoughts [25] regarding diversity in their curriculum and feelings of inclusion and exclusion in their education. The data collected for this research are part of a larger project conducted between three faculties of Utrecht University, the Netherlands, aimed at developing a reflection tool to evaluate the extent to which diversity and inclusion are part of the curriculum and developing interventions to foster diversity and inclusion within the curriculum.

### Study sample and procedure

A combination of convenience, purposive, and snowball sampling was used [26]. First, we used convenience sampling to recruit students to ensure a sufficient number of participants and to give students with different characteristics the opportunity to participate. Due to the COVID-19 pandemic, students were mainly recruited online by distributing flyers and messages via various channels, such as a posting on the faculty’s announcement page on the electronic learning management system and on the study program’s newsletter. The focus groups were also announced in some (online or face-to-face) lectures. Second, we used purposive sampling to ensure a representation of students from different diversity groups [27, 28]. Students were approached through a platform at UMC Utrecht that works on Diversity and Inclusion within education. Given the sensitivity of the subject, snowball sampling was used by asking participating students whether they knew other students who might be interested in the study [29].

The focus groups were conducted online via Microsoft Teams in 2021, took 60–90 min, and were audio and video recorded. The focus groups were led by one moderator and one or two additional moderator(s) asked additional questions and took notes. The moderators did not know the participants and did not have a direct formal role (e.g., teacher, supervisor) in their education. The moderators all had knowledge of the research topic. The focus group conversations were based on a semi-structured interview guide (see Appendix A). To ensure coverage of different topics, this guide included questions regarding 1. students’ definition of diversity and an inclusive learning environment, 2. positive and/or negative experiences regarding diversity in their education, and 3. feelings and experiences of inclusion and exclusion. To elicit participants’ opinions and experiences, various techniques were used, such as probing around key questions, paraphrasing their answers, seeking divergent opinions, and stimulating discussion among participants [30].

To create a safe environment and to make it as comfortable as possible for participants to share their experiences, several measures were taken. First, an introduction round was conducted to increase familiarity with each other. The moderator also stressed at the start of each focus group that there were no right or wrong answers, that the researchers were interested in the personal views of the students, that it is fine if students differ or disagree, and that the conversation in the focus group would remain confidentially [30, 31]. Special attention was paid to the order of the discussed topics [32]; more neutral topics (e.g., defining diversity) were discussed before more sensitive topics (e.g., students’ personal experiences).

Participants received a €10 voucher as compensation for their participation, which was internally funded. Written informed consent was obtained from the participants in advance. Participation was voluntary. Ethical approval for this study was obtained from the local Institutional Review Board (file no. 21–0070).

### Data analysis

The recordings of the focus groups were transcribed verbatim. In the transcriptions, the researchers made a note of gestures and tone of the participants. These were not analyzed separately, but added to the interpretation of students’ statements. Thematic analysis was used to code and analyze the data, identify common themes, topics, and meaningful patterns [33]. First, they individually read the transcripts multiple times to get a feeling for the depth of the data and then together discussed ideas that emerged. After collaboratively coding part of the data to establish a basic coding scheme, the first and second author coded all data independently using Nvivo v. 12.6. Next, the researchers discussed their coding system until consensus was reached. Together, the researchers then scrutinized and organized the resulting codes into themes. Each theme consisted of conceptually similar or related codes. Data collection and analysis were conducted in an iterative way. To establish trustworthiness of our findings, we established coherence by checking the data so that there were no internal conflicts or contradictions, used investigator triangulation in coding the data, (partly) used purposive sampling, and collected and reported thick descriptive data [34]. The themes and codes, a description of the codes, and a sample for each code can be found in Appendix B. An edited transcription was used in the quotes for the Results section to make the quotes easy to read and to ensure the core message of each respondent was clear.

## Results

Four focus groups, consisting of three to nine students (20 students in total) from the Faculty of Medicine participated in this study. Of the participants, seven were bachelors' students and 13 were masters' students. Two out of the 20 students were international students from outside of Europe, five students had non-dominant ethnic background (including Turkish, Moroccan and Surinam), one student identified as non-binary and queer, four students had non-dominant sexual orientation (three being homosexual, one being lesbian), and one student indicated having autism.

**Fig. 1:**
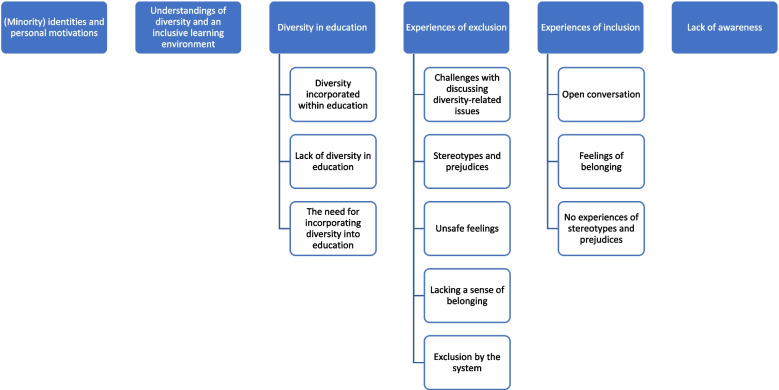
Overview of themes and subthemes

There were six main themes that emerged from the focus group discussions: (1) (Minority) identities and personal motivations, (2) Understanding of diversity and an inclusive learning environment, (3) Diversity in education, (4) Experiences of exclusion, (5) Experiences of inclusion, and (6) Lack of awareness. These themes and their codes are presented in Fig. 1 and further described below.

### (Minority) Identities and personal motivations

Students were asked to introduce themselves and to share their motivation to participate in the focus group. Although participants were not obliged to share information about their gender, sexual orientation, age, nationality, ethnic background, religious background, socioeconomic status, mental/physical disabilities, or other characteristics, many chose to share this information. This highlighted the diverse sample of participants. During the self-descriptions, students also chose to share personal experiences and opinions: “And when people realize I am Turkish, I often get: ‘Oh, but you don’t look Turkish at all.’ And then the next question is, what do Turkish people look like” (participant 20), “I’m gay myself, I have a very nice boyfriend and that’s where diversity starts for me because I think that is how I, as a White man, differ from the average White man” (participant 12).

In addition, some of the students explained their personal motivation to work on incorporating diversity and inclusion (more) within their program and why they found it important to participate in this study: “[…] there are more people who don’t necessarily look like me, but I want to provide good care to them” (participant 14), “[…] yeah, and that is the reason I think that I am present here, because I find it really interesting and very important and I am curious as to how we can improve that” (participant 16).

### Understanding of diversity and an inclusive learning environment

When asked to define diversity and inclusive learning environment, the participants made no distinction between the two terms and provided broad definitions which included aspects such as culture, ethnic background, mental/physical disabilities, sexual orientation, socioeconomic status, and ideas/perspectives: “Recognizing and also respecting different cultures” (participant 2), “[…] yes, I think it is actually very broad. So, I think it has to do with that there are differences, and that those differences can be there, whether that's in origin or in sexual preference, or in interests” (participant 16), “Diversity to me I think is that there are differences, everyone is different, but that shouldn’t matter. It is present and that is something beautiful. So that is diversity to me” (participant 15).

Students also mentioned in their definitions that everyone should be considered, able to participate, have equal opportunities, be respected, and everyone should feel they belong. This is illustrated in the following quotes: “To me diversity is simply that everyone is allowed to participate and that everyone is participating, and all differences are taken into account” (participant 19), “Inclusion is that all those people are welcomed and heard” (participant 6).

Several students defined diversity and inclusion separately, for example: “Definition of diversity is maybe that there is indeed a bit of a reflection of the population, which you can see in your study program. And especially inclusion, that you feel comfortable in your studies, that you feel at home” (participant 10).

### Diversity in education

The diversity in education theme was divided into three codes: (1) diversity incorporated within education, (2) lack of diversity in education, (3) the need for incorporating diversity into education.

#### Diversity incorporated within education

Some students gave examples of how diversity was already included in their education. These examples were seen by the students as important and helped raise awareness: “I did have (class name) recently, one of the last [sessions] was entirely about diversity […]. It was about what your blind spot is, how you can handle that and little about self-awareness, so there is some attention given to it” (participant 10). “We are also learning a bit about religion. How you, for example, start a conversation with someone from a particular religion […] that made me think” (participant 11).

#### Lack of diversity in education

One of the most common topics students addressed was their experiences with and views on the lack of diversity in education. Students indicated that they frequently miss information in their education about “non-Dutch culture” and “people not looking like them” for whom they also want to provide good care. This is lacking in multiple aspects of their study program: in (1) research and research-based knowledge, (2), the content itself, and (3) the context. Research and research-based knowledge refers to the lack of diversity in research populations and research topics students learn about in their medical education, for example a student said: “I do kind of miss the recognition that most research in the Western world is really done on a typical male and White population.”

The content of the medical curriculum was evident in examples of a lack of knowledge about communication with patients from different cultures, and a lack of diversity in lectures cases and learning materials. One student (participant 1) stated “that there are always a lot of pictures of light-skinned people and not with white-skinned people and for example, a lot of pictures have ‘pale skin’, and you can’t see that on a person of color.” More broadly, participants referred to a lack of diversity in the “medical world,” in patient care, and a lack of knowledge about diversity of physicians and teachers. For example, one student (participant 14) expressed that there are more than “only White straight men” in medicine and that this is missing in medical education. Regarding the knowledge of diversity among physicians, one student (participant 20) said: “I am missing role models. Right now, in my clinical internship I am looking for a physician who is a good role model for me, […] who does pay attention to ‘he or she’, the right naming for the right patient. I miss that and in education, where you have the feeling that you don’t fit in and that you have to add to it.”

Students perceived the context about diversity lacking in the student and teacher population and language in both written and verbal communication not always as inclusive. Regarding language use, some students expressed that their study material sometimes perpetuates stereotyping: “Recently, I had a lecture about ‘the Eskimo people’. You cannot say that. […] that is also a swear word for those people” (participant 2). This student went on to suggest that study material should be more inclusive, for example use language that does not insult or offend people based on their diversity identity/background.

#### The need for incorporating diversity into education

Next to describing their views on the lack of diversity in medical education, students often talked about the need to incorporate diversity into their education and how this could be done, especially in relation to the medical curriculum. Most students indicated that diversity topics, such as gender, sexuality, religion, and ethnicity, which are important in the medical field, need to be discussed more (openly). One student (participant 17) mentioned that while the patient population is diverse, most medical students are white. They went on to explain that this makes incorporating diversity in education even more relevant and could have a positive influence on patient care as well as their development as health professionals. Other students also mentioned that it is important that they gain the necessary knowledge during their education to provide quality care to a diverse patient population. According to the participants, this can be done by including the following: “[…] a wider range of simulation patients, broader cases, not those stereotypes, but also: […] how do I communicate for example with a patient with autism” (participant 17). Other aspects mentioned in addition to cases and learning to communicate with a diverse patient population were being transparent about which populations research is done and what is lacking in research. Having a more diverse physician population, incorporating knowledge needed to deal with a diverse patient population such as definitions of gender and sex, differences in prevalence of illnesses by ethnicity, and a focus on different skin tones rather than only white skin were mentioned.

In one of the focus groups, students discussed that regular lectures on diversity during the academic year would be a good idea: “[…] a lecture about ‘what is diversity and inclusion’ and measure students’ reactions because […] until yesterday I was focused on gender and sexuality. And I know for sure more people are very focused on this, even though it is much broader. And that you start with that [referring to the topic diversity and inclusion], and you discuss it more often and go more in depth on it” (participant 7).

### Experiences of exclusion

Different codes of feeling excluded or observing exclusion emerged from students’ responses in the focus group discussions. The codes belonging to this theme are: (1) challenges with discussing diversity-related issues, (2) stereotypes and prejudices, (3) unsafe feelings, (4) no feelings of belonging, and (5) exclusion by the system.

#### Challenges with discussing diversity-related issues

Some students expressed that it is not always easy or possible for them to discuss diversity-related issues (e.g., diversity lacking in education, exclusive language use). This was due to several reasons, such as the expectation of negative reactions, finding the issue too insignificant to address, the hierarchical relationship with a teacher, or because they felt unsafe addressing an issue from their own background. Examples of the latter two reasons are: “I also don’t want to correct a professor now or sound like a know-it-all and annoying” (participant 2). A student (participant 12) commented on a joke related to his sexual orientation made by a physician: “I actually didn’t dare [to speak up] to be honest. One time, I can remember that a physician of the surgery, but it wasn’t my direct mentor [had made a comment], but still I thought ‘I don’t know you well and you don’t know me’, and you know it often happens so quickly that you think you are too late to react in the moment, and to come back to it later. I don’t feel completely safe with that. Can I say this, actually, I have to do it, but I just don’t dare to, but sometimes I think I really want to.”

Students also mentioned they carefully consider when addressing an issue and whether it is worth addressing: “I do notice that I often find it hard when I hear something in a class or lecture. I don’t know if I have the guts to go to the lecturer and say hey, I notice that you said this in a certain way. I am tired of having the conversation again or being the ‘angry Black girl’ who says hey, I am going to tell you that I find it racist. I notice that I find it very difficult” (participant 6). “[In some cases] I go against it, because I think, this must be corrected, why do you just assume this. But in other moments I don’t, it really depends on the context” (participant 17).

#### Stereotypes and prejudices

Most students mentioned experiencing exclusion related to encountering stereotypes and/or prejudices. This included both being stereotyped themselves and experiences where they saw or heard others being stereotyped in the education environment. For example, one student (participant 17) shared an experience during a clinical internship when others found out they had a Turkish background: “They also always assume I am a Muslim, which I think is very peculiar because I don’t know why they assume that. But you are some kind of stereotype in the eyes of people, and I think that is a shame, and also painful.”

The participants explained that stereotyping not only happens by patients but also by fellow students and physicians. They also mentioned educational material or information in lectures perpetuated stereotypes: “What bothers me a bit I think, is that for example homosexual men are [categorized] as… having HIV (human immunodeficiency viruses) and are very high risk of STIs (sexually transmitted infections). [In lectures] they immediately say, if someone is homosexual, let’s try all the tests on this person […]” (participant 8).

#### Unsafe feelings

Different students expressed having unsafe feelings in the learning environment, mostly due to remarks or statements by others. One student shared an experience where someone else had made a joke about homosexuality: “[…] it sometimes gives me a little uncomfortable feeling, an unsafe feeling, even though it wasn’t directly aimed at me […]” (participant 12). Another mentioned: “I was doing my clinical internships and I walked into the operating room… and the first comment I get there is that I was very in proportion with my, my very beautiful model body and it was a shame that there was such a wide operating suit over it [referring to her body] … and that they preferred to see me in a different way… and that was said by a surgeon in a very male team [voice breaks]. Well, okay, not very safe I guess… then I felt really threatened” (participant 8).

#### Lacking a sense of belonging

Some students expressed that they (sometimes) did not feel they belonged in the learning environment: “I don’t feel super at home at the university. I have the feeling Medicine is a pretty… conservative surrounding… while they don’t have the feeling like they are conservative themselves, that’s the hard part […]” (participant 18).

Some students described lacking a sense of belonging because of their minority identity, for example as international students or when having different ideas/perspectives. Other students said this was due to the culture on their program or in the medical environment which they described as “conservative” (participant 18) and “not always a pleasant atmosphere” (participant 8).

#### Exclusion by the system

Students mentioned several aspects of the education system they perceived as exclusive, including assessment and the selection procedure. This included both personal experiences with exclusion in the system and students’ observations of the system without necessarily having personal experiences with it. A few students mentioned difficulty with the language used in exams for students with a non-dominant ethnic background. One student explained that: “Yeah, I was of course born and raised here; but bilingual. […] There are still some words which I for example don’t know during the exam, … and I find it kind of awkward to ask, I don’t know what this means” (participant 2).

Regarding selection, students shared their ideas about how both training for selection and the content they are being assessed on are exclusive: “There are a lot of courses offered for the selection and you must pay for those. I don’t remember how much it was precisely, but it goes up from €100 euros to a few hundred euros, and I think there was a difference for people coming from a low-income family or who cannot afford it. This then creates a barrier to getting into medical school because [those that take the courses] have a better chance at making it in [medical school]” (participant 4). “[…] I took part in one of those courses where you get intensive training, you get summaries, learning material… and all kinds of exam material. So, if you can’t afford it, you’re actually one step behind those who can afford it” (participant 2).

### Experiences of inclusion

Students also described experiences of inclusion. These were classified as: (1) open conversation, (2) feelings of belonging, and (3) no experiences of stereotypes and prejudices.

#### Open conversation

Students shared experiences of having an open conversation with peers and/or teachers about diversity-related topics. This was appreciated and seen as important, helped reduce prejudices, and stimulated understanding: “So, [fellow students] say: ‘Hey, do you have to wear that headscarf because of your parents or how does it work?’ Then you explain how it works and then a very open conversation arises. While I like that they ask those questions, even if they have a certain prejudice in the beginning, because when they asked the question and I provide my input, a certain understanding arises, and then you have eliminated that prejudice a little more” (participant 19).

#### Feelings of belonging

A few students indicated experiencing a sense of belonging on their program. This included feeling accepted within the learning environment or when they felt part of a group. “I actually feel very much at home on the program because I think it is a very nice place to be” (participant 12). “[…] I do feel at home in my studies and such” (participant 10).

#### No experiences of stereotypes and prejudices

Some of the students stated that they had no experiences or observations of prejudices and stereotypes: “But I entirely agree with [name of another participant], that I myself don’t experience prejudice, but I find it quite annoying that apparently it happens very often” (participant 16). “So, in the first sense I have never experienced these kinds of things” (participant 15). “Well, I have to say that from fellow students, when I'm in conversations with them, that I don't necessarily notice annoying prejudices” (participant 19).

### Lack of awareness

Lack of awareness was stated many times in relation to the earlier described themes. Multiple students expressed that they believe physicians lack awareness about diversity-related topics, such as the cultural background of ethnic minorities. They explained that by taking these issues into account and increasing awareness, physicians will be better prepared to provide care to a diverse patient population: “And those are little things that happen consciously or unconsciously, where physicians are or are not aware of. So, I think that we can give the students some more tools for the future. And with that we get physicians who can take that into account, can recognize it, and are able to implement it in practice a little better” (participant 19).

The consequence of lack of awareness of the differences in mastery of the Dutch language present in the student population was shared by one student: “What I thought of the clinical internships was, when you seem a bit in doubt, [people assume] you don’t have the proper knowledge. […] It can be that someone is shy or in my case I do speak Dutch, but it is not my first language. […] Sometimes I need time to process information before I can say something useful. That is something I would like to see better [more awareness]” (participant 9).

## Discussion

In this focus group study, we explored students’ views on, and experiences related to diversity and inclusion in medical education. We also explored their experiences of inclusion and exclusion in their study program. These insights can help to create a more inclusive educational environment and ensure the medical curriculum aligns with the diversity that is present in our society. Our study showed that students perceive a lack of diversity in their education and articulated that more diversity education should be incorporated in the medical program. Students described their own and others’ experiences of exclusion in education although experiences of inclusion were also shared. The lack of awareness about diversity among teachers, health care professionals, and (fellow) students appears to be an important reason for a lack of diversity and exclusion experiences in medical education. This has implications for the content and design of the medical curriculum in which diversity and raising awareness for diversity should be integrated more.

### Diversity in education

Medical students feel they are not being sufficiently prepared to serve a diverse patient population. Students emphasized there is a lack of diversity within medical education, are convinced that more diversity needs to be incorporated into the curriculum and are personally motivated to contribute to this. There were several areas within the curriculum mentioned that lack diversity: (1) research and research-based knowledge (i.e., research topics, research populations), (2) the content itself (e.g., cases, communication with culturally diverse patients), and (3) the context (e.g., the teacher and student population, inclusive language). Students’ perceptions of a lack of diversity in education is consistent with prior research that showed there is an unmet need within traditional medical curricula concerning the topics of diversity, health disparities, and health equity [35, 36]. Our findings resemble that of Muntinga et al. (2016) who point to a lack of diversity in the content of the medical curriculum in the areas of patient-physician communication (e.g., communicating with diverse patients in terms of gender, class, and culture), developing medical knowledge and skills (e.g., diagnosing diverse patients, disease prevalence in diverse patients), and reflection on students’ and health care professionals’ own awareness, biases, and assumptions [36]. Our study also aligns with research that showed students perceive gaps in the medical curriculum related to diversity issues [11–13]. The notion of social responsibility points to the importance of medical educators preparing students to serve a diverse society to help eliminating disparities and create socially responsible health care professionals. However, our findings indicate that medical education is limited in this regard. Despite limitations in the curriculum, our findings also showed students felt social responsibility as they were motivated to pay attention to diversity and incorporating it into medical education.

In addition to a lack of diversity education in the medical curriculum, participants also observed that if attention is paid to diversity in the patient population, this is often in a stereotyping manner. This aligns with the recommendation of Lim et al. (2021) to assess and update educational material to remove stereotypes and ensure information is grounded in evidence-based medicine [37]. Other ideas participants shared were increasing diversity among the health care professional population, incorporating diversity in cases, and using more inclusive language. The few examples students shared of diversity present in their education is promising because they help students to become more aware of diversity issues.

### Inclusion and exclusion experiences

Students also shared personal experiences of both exclusion and inclusion within their program. Although it is encouraging that diverse students shared positive experiences of open conversations [38] and feeling they belong on their program, there were also multiple instances of exclusion. Students described experiencing and observing stereotypes and prejudices related to their (minority) identities, such as ethnic background and sexual orientation. Not all students felt safe all the time, and some did not (always) feel they belong on their program. An absence of feelings of belonging could lead to exhaustion, isolation, emotional distress, and health problems [16, 39–41]. Also, the way the education system is organized leads in some ways to observations and experiences of exclusion.

A possible reason for students, particularly students identifying with a minority identity, to feel excluded is the lack of diversity in the medical curriculum. This was implied in statements by several participants in this study. Likewise, Hurtado and Ponjuan (2005) and Nuñez (2009) found that students had a stronger sense of belonging when diversity was incorporated in the curriculum [42, 43]. Students also referred to a lack of awareness among teachers, health care professionals, and (fellow) students about diversity and its importance for creating an inclusive learning environment and delivering the best quality of care to a diverse patient population. Possibly, increasing awareness could be an important first step for incorporating diversity in education and for reducing exclusion experiences of students.

### Strengths and limitations

Due to COVID-19 restrictions, data was collected online. This made it less possible to observe all non-verbal signs of the participants [44]. However, we were able to observe facial and voice tone in addition to some bodily movements and gestures.

These restrictions also limited our participant sample. While the demographic of the group was quite broad, it is not a complete representation of the higher education student population. Most participants identified themselves with a minority identity and/or were interested in diversity-related topics. Therefore, they were personally motivated to work towards increasing diversity in the medical education program. If a larger and more diverse population was observed, there is a possibility that some students would be less interested in incorporating diversity-aspects in education.

By not requiring participants to share specific information about their background characteristics, some areas in the data collect are incomplete. However, most participants shared personal information which made it possible to characterize our sample and showed that diversity in ethnic background, nationality, gender, sexual orientation, and more was present among our participants. Having a diverse collection of participants also helped create a safe environment for participants to disclose information about themselves. This led to more meaningful insights into the topic where participants felt safe to share their experiences and explain their thoughts, increasing the richness of the data.

### Recommendations

Our findings showed that students experience a lack of diversity in education and that more could be incorporated into medical education. Based on our findings, we generated recommendations from the themes that most clearly pointed to practical implications:

- Diversity in education: Diversity-related aspects should be included (more) in study materials, such as showing different types of skin colors in medical pictures and presenting more diversity in case studies. This will better prepare students to serve a diverse patient population. It is the social responsibility of the institution to provide teachers with time to re-examine and redesign the curriculum in this respect.
- Diversity in education: More attention should be paid to inclusive language in education. For example, language used in study materials and by educators should not offend people. Therefore, more awareness and education should be provided on inclusive language for teachers, physicians, and students.
- Diversity in education: When discussing the prevalence of diseases with patients with a minority identity, social contextualization should be provided. For example, when discussing the higher prevalence of HIV in homosexual men, the context should be given in order to prevent stereotyping [37].
- Experiences of exclusion: Tools or guidance about how to handle exclusion situations as an active bystander and to build and strengthen their resilience should be provided to students. This can be useful during encounters with patients in clinical internships.
- Experiences of exclusion: Assessments (and learning materials) should also be understandable for non-native/bilingual students. Teachers/assessors should check how understandable the assessments are for these students. This can be done by having the assessments checked by non-native/bilingual colleagues or students. New and difficult words should be explained in diverse ways to make it understandable for these students.
- Experiences of inclusion: Open conversations about diversity-related topics among students, teachers, and between students and teachers should be supported. This can be introduced in the classroom to help reduce prejudices and can stimulate understanding and learning.
- Lack of awareness: Teachers and physicians should receive awareness training on culture and gender diversity. This can also be introduced throughout the curriculum.

### Future research

The findings of this study reveal opportunities for future research. For instance, research into knowledge about how diversity-related aspects can it be incorporated in existing curricula is needed. This should include how cases can be made more diverse without stereotyping and how to incorporate inclusive language use more in medicine without giving teachers, health care professionals, and/or students the feeling of being censored. In addition, research is needed into the importance of sense of belonging within medical education and its relationship with students’ perceptions of how diversity is incorporated in medical education.

Considering this study mainly focused on knowledge in curricula, future research could potentially focus on attitudes, which are important to develop students’ sense of social responsibility as health care professionals. While this study only focused on student perspectives, future research could also examine teachers’ perceptions to investigate how they might differ from their students.

## Conclusion

The present study provided in-depth descriptions of the experiences and views of a diverse group of students on diversity within medical education and inclusion and exclusion experiences. Students perceive that diversity is lacking within medical education and if diversity is present in the curriculum, this is often stereotyping. Also, they frequently experience or observe exclusion in their program. Medical educators have the social responsibility to educate their students to be able to serve the needs of a diverse patient population and to ensure all students feel included and at home in their medical education program. Eliminating inequality of opportunities for medical students as well as disparities in health care should be the goal of all medical universities. Patient populations will continue to become more diverse. Therefore, medical universities must prepare their students for this ever-changing world.

## Supplementary Information

**Additional file 1:** **Appendix A.** Interview Guideline Focus Groups

**Additional file 2:** **Appendix B.** Codes, Description of Codes, and Sample Codes

## Data Availability

Anonymized data are available from the corresponding author on request.

## Abbreviations

UMCU: University Medical Center Utrecht
COVID-19: Coronavirus disease 2019
HIV: Human immunodeficiency viruses
STIs: Sexually transmitted infections
