# Supplementary material for: Inclusion and diversity within medical education: a focus group study of students’ experiences
Source: BMC Med Educ. 2023 Jan 25;23:61. doi: 10.1186/s12909-023-04036-3 (PMC9875758; doi:10.1186/s12909-023-04036-3)
Supplement: Supplementary file 1 — Additional file 1: Appendix A. Interview Guideline Focus Groups [file 12909_2023_4036_MOESM1_ESM.docx]

Appendix A

Interview Guideline Focus Groups

1. Could you introduce yourself and share something about your motivation to participate in this focus group?
2. What do you think diversity and inclusion entail?
   1. How would you define diversity and inclusion?
   2. What does an inclusive learning environment mean to you?
3. To what extent is diversity incorporated in your study program’s curriculum)?
   1. To what extent is diversity part of different aspects in the curriculum (i.e.., learning goals, literature/other materials, cases, assessment, clinical internships)?
   2. Which tools does your program use to attain skills to deal with diversity?
   3. How can diversity be integrated (more) in the curriculum?
4. What (positive and/or negative) experiences of inclusion or exclusion did you have in your study program?
   1. Do you have any of those experiences in the contact with teachers (e.g., experiencing prejudices, unsafety)?
   2. Do you have any of those experiences in the contact with fellow students (e.g., do you feel excluded during group assignments, do you feel like being a ‘minority’)?
   3. Do you feel you belong at your study program and/or the university?
   4. How can inclusion be stimulated in your education?
   5. What does the ideal inclusive curriculum look like for your study program?
5. Is there anything else you would like to share?
