# Supplementary material for: Inclusion and diversity within medical education: a focus group study of students’ experiences
Source: BMC Med Educ. 2023 Jan 25;23:61. doi: 10.1186/s12909-023-04036-3 (PMC9875758; doi:10.1186/s12909-023-04036-3)
Supplement: Supplementary file 2 — Additional file 2: Appendix B. Codes, Description of Codes, and Sample Codes [file 12909_2023_4036_MOESM2_ESM.docx]

Appendix B

*Codes, Description of Codes, and Sample Codes*

| Themes & codes | Description | Sample |
| --- | --- | --- |
| (Minority) Identity | | |
| Ethnic (minority) identity | Students’ reference to their own ethnic background. | “So then you would need to explain that you have a Turkish background” |
| Gender identity | This could entail either a description about students’ gender or their sexual orientation. | “Um, well I’m gay myself, I have a very nice boyfriend. Uhm, and that’s where diversity starts for me because I think that’s how I, as a White man, differ from the average White man.” |
| Identity and disabilities | A description of students’ mental or physical disability. | “uh…. And I am also autistic” |
| Nationality and identity | A description of students’ nationality. | “… I think I’m the only international student.” |
| Definition of diversity and/or inclusive learning environment | This includes students’ views on how they define ‘diversity’, ‘inclusion’, and/or an ‘inclusive learning environment’. | “Diversity to me means a kind of, yeah it doesn’t have to be a reflection of society per se, more kind of that you have variety in ideas, in the people you see, that you encounter on a daily basis and inclusion is more of are the diverse people able to be active or participate to the extent that they would like to.” |
| Diversity in education | | |
| Diversity incorporated within education | Students describe how diversity is incorporated in their study program. | “Uhm… we’re learning a little bit now about religions how you, for example, start the conversation with someone with a religion. …” |
| Lack of diversity in education | Students express a lack of diversity in (different aspects of) their study program. | “but I miss in education, uhm and especially in communication education, uhm some things in that. Like hey, how do you handle, how do you perform a sexual anamnesis on uh a young homosexual man in the emergency room? Um… I really miss those kinds of things.” |
| Lack of awareness | Students’ descriptions of experiences of a lack of awareness of the importance of diversity and their views on the importance of this awareness. | “I, I… I’m also sure that no uh harm is intended, but more like people don’t know and in that way they hurt people w….without knowing it.’ |
| Need for incorporating diversity in education | Students mention the need to incorporate diversity in their study program and/or diversity aspects they think should be incorporated. | "So I would just really like us to, for example, have a lecture on this, a lecture on gender and sex, and a lecture on background, and how to name that. If you just state that properly from the beginning, then it's way easier to build on that in subsequent... things and then you can continue to integrate that..." |
| Experiences of exclusion | | |
| Challenges with discussing diversity-related issues | The extent to which students describe they experience challenges with discussing diversity-related topics/experiences and specifically experiences of exclusion. | "Yes... during clinical internships you have to be careful with that, um, then you really have to feel of um, here I have to do it, here I don't have to do it. Because that um, practice is really, that can kill you. Criticizing a doctor can really cost you your head, so you really have to start supplementing... feeling." |
| No feeling of belonging | Students’ descriptions of experience a lack of belonging to their study program. | “Uhm, yeah I moved to the Netherlands for my Medicine degree. So yeah there have been moments I didn’t really feel at home. And yeah already since the introduction week uhm… so… they are uhm involving Dutch students. And… I think that in my year I am the only foreign student. And... yeah I… don’t feel involved at a lot of moments.” |
| Feeling of unsafety | Students’ experiences of feeling unsafe or uncomfortable during their study. | “And.. it sometimes gives a little a uh, uh … a little uncomfortable feeling, an unsafe feeling, uhm even though it wasn’t directly aimed at me, it never has been.” |
| Stereotyping | Students’ experiences with or observations of stereotyping within medical education. | “Yeah so, for that matter I think especially many, at many specialisms also not, but that you are still looked upon as woman like ‘what are you doing here?’ […] And particularly when you maybe, yeah, yeah I don’t know, that, that you look a certain way, that you get, uhm.. yeah also, also a, a stereotype thro, thrown over you.” |
| Exclusion by the system | Students’ descriptions of how they perceive different aspects of their study program as not being inclusive for all students. | “Someone who would love to do medicine but lives in straitened circumstances, they aren’t going to do a course of, well what is it, 120 euros, maybe? If it is not more […] I thought, remember that I was really occupied with it back then, that I thought, this I think is really… and then I came to the program and it turned out that almost everyone I spoke to, did that cursus. Then I thought, well, wow I have been lucky for getting in at all… That that cursus was like a golden ticket to get uh in here.” |
| Inclusion | | |
| No prejudices | Students’ descriptions of not experiencing any prejudices. | “Well I have to say that I from uh fellow students, when I am in conversations with them, wh, that I don’t necessarily notice bad prejudices” |
| Open conversation | Students’ experiences of or views on the importance of an open conversation. | “So uhh that they say: ‘Hey, do you have to wear that headscarf because of uh your parents or how does it work?’ Then you explain how it works and then… a really open conversation arises. Uhm, while, I do find it great that they ask those questions, although they sometimes had a certain prejudice in the beginning, because when they asked the question and I gave my input, well than, than you get a, that there uh a certain understanding is created” |
| Feelings of belonging | Students’ descriptions of feeling they belong at their study program. | “Uhm… and I actually feel very much at home at the program because I think it is a very nice place to be” |
| Personal motivation | Students’ descriptions of their personal motivation to work on and of the importance of an inclusive environment and diverse curriculum at their study program. | “That there are more people who don’t necessarily look like me, but that I want to provide good care to them.” |
